# A Hidratação Oral pode ser uma Opção na Prevenção da Nefropatia Induzida por Contraste em Intervenções Coronárias Eletivas

**DOI:** 10.36660/abc.20220868

**Published:** 2023-02-13

**Authors:** Paulo Roberto Barbosa Evora

**Affiliations:** 1 Universidade de São Paulo Faculdade de Medicina de Ribeirão Preto Ribeirão Preto SP Brasil Universidade de São Paulo – Faculdade de Medicina de Ribeirão Preto – Cirurgia e Anatomia, Ribeirão Preto, SP – Brasil

**Keywords:** Hidratação/métodos, Nefropatias/prevenção e controle, Hidratação Oral, Hidratação Intravenosa, Nicorandil/uso terapêutico, Cateterismo Cardíaco/métodos

O estudo^1^ tem perfil editorial questionável dos ABC Cardiol e não apresenta nenhuma novidade. No entanto, sua decisão de publicação é útil para cardiologistas em sua atividade de controle pré-operatório. Sempre se fala em insuficiência renal aguda, e às vezes tem-se a impressão de que está sendo subestimada ou superestimada.

Esses protocolos incluem 1) Medicamentos; 2) alcalinização com bicarbonato de sódio, e; 3) hidratação. Dentre as diversas drogas, o nicorandil tem sido utilizado em pacientes com disfunção renal crônica. Um estudo realizado para avaliar seu efeito preventivo em pacientes de alto risco revelou eficácia substancial em relação ao protocolo de hidratação para cateterismo cardíaco.^2 , 3^ Dentre as diversas drogas, o nicorandil tem sido utilizado em pacientes com disfunção renal crônica.^4 , 5^

Concordamos com os autores^1^ que a medida preventiva mais eficaz até o momento é a hidratação intravenosa (IV). No entanto, as evidências atuais mostraram que a hidratação oral (HO) é comparável à infusão intravenosa, mas a sua aplicação ambulatorial ainda carece de mais investigações em procedimentos coronarianos eletivos.

O presente protocolo de HO parece ser semelhante ao protocolo de IV hospitalar na proteção renal. Os autores sugerem testá-lo em grandes ensaios na tentativa de provar que a HO é eficaz e seria de fácil adoção na prática médica.
