# Supplementary material for: A Hidratação Oral pode ser uma Opção na Prevenção da Nefropatia Induzida por Contraste em Intervenções Coronárias Eletivas
Source: Arq Bras Cardiol. 2023 Feb 13;120(2):e20220868. [Article in Portuguese] doi: 10.36660/abc.20220868 (PMC10263461; doi:10.36660/abc.20220868)
Supplement: Translation

# Translation: Oral Hydration May Be an Option to Prevent Contrast-Induced Nephropathy in Elective Coronary Interventions

**Authors:** Paulo Roberto Barbosa Evora

**Affiliations:** 1 Brasil Universidade de São Paulo – Faculdade de Medicina de Ribeirão Preto – Cirurgia e Anatomia, Ribeirão Preto, SP – Brasil

**Keywords:** Fluid Therapy/methods, Nephropathies/prevention and control, Oral Hidratation, Intravenous Hidratation, Nicorandil/therapeutic use, Cardiac Catheterization/methods

The study^1^has a questionable ABC Cardio editorial profile and does not present any novelty. However, its publication decision would be useful for cardiologists in their preoperative control activity. Acute renal insufficiency is always discussed, and sometimes people say it is underestimated or overestimated.

Many studies support alternative protocols to prevent contrast-induced nephropathy (CIN) caused by iodine contrasts. It is opportune mentioning the safety of ambulatory protocols for vessels and heart exams carried out on the ambulatory “cath-lab.”

These protocols include 1) Drugs, 2) alkalinization with sodium bicarbonate, and; 3) hydration. Among different drugs, nicorandil has been used in patients with chronic renal dysfunction. A trial to assess its preventive effect on high-risk patients revealed substantial efficacy over hydration protocol for cardiac catheterization.^2 , 3^Among different drugs, nicorandil has been used in patients with chronic renal dysfunction.^4 , 5^

We agree with the authors^1^that the most effective preventive measure to date is intravenous hydration (IVH). However, current evidence shows that oral hydration (OH) is comparable to intravenous infusion. Nevertheless, the effectiveness of ambulatory OH still needs more investigations in elective coronary procedures.

The OH protocol performed by the patient appears to be like the hospital IVH protocol in renal protection. The authors suggest testing it in large trials in an attempt to prove that OH is effective and would be useful in medical practice.
